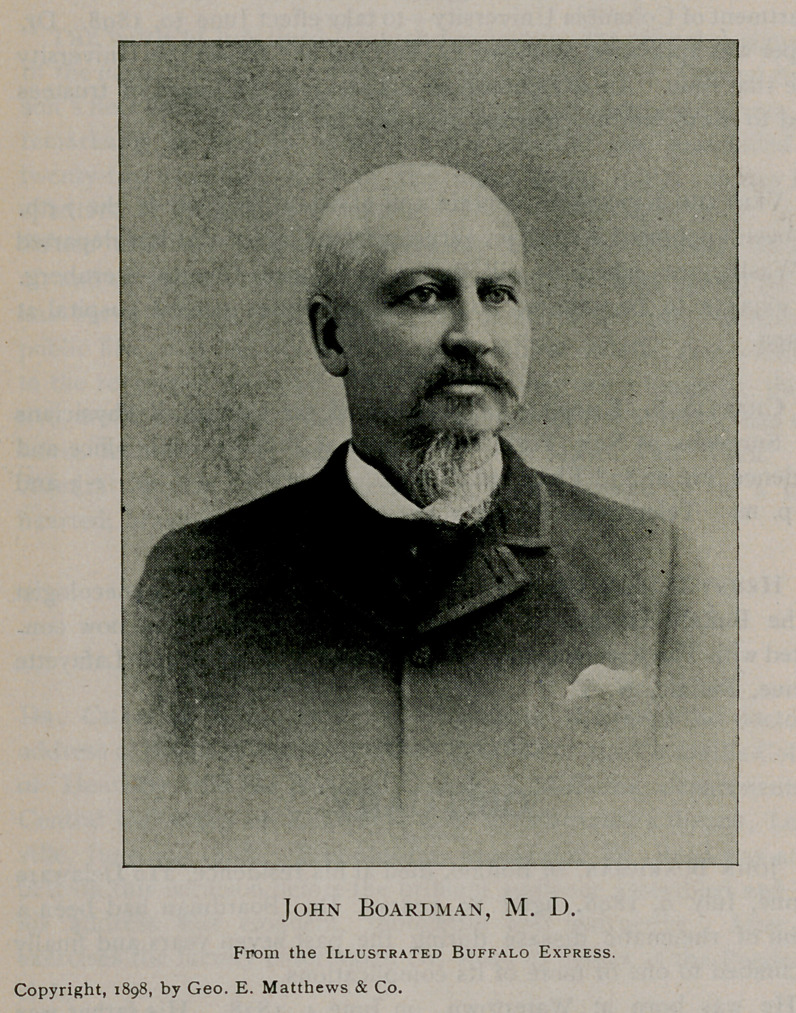# Dr. John Boardman

**Published:** 1898-08

**Authors:** 


### OBITUARY.

Dr. John Boardman, of Buffalo, died at his residence, 210 Delaware avenue, July 9, 1898, aged 70 years. Dr. Boardman had been a victim of rheumatic disease during the past seven years and finally succumbed to one or more of its complications.

He was born at Watertown, on June 4, 1828. His father was Rev. George S. Boardman, D. D., who died at Cazenovia in 1877. The younger Boardman was graduated from Williams College in the class of '49, attended two courses of study in the medical college of the Buffalo University as a student of Dr. Frank Hastings Hamilton and was graduated from the medical department of the University of Pennsylvania in 1853.

He was appointed demonstrator of anatomy in the medical department of the University of Buffalo in 1854, in which capacity he served until 1858, when he resigned ; from 1854 to 1858 he was physician to the Buffalo Orphan Asylum and was attending surgeon to the Buffalo Hospital of the Sisters of Charity from 1856 to 1873. In

JOHN BOARDMAN, M. D.

From the ILLUSTRATED BUFFALO EXPRESS.

Copyright, 1898, by Geo. E. Matthews & Co.

1883, Gov. Cleveland appointed him a member of the board of managers of the Buffalo State Hospital for the Insane, a position he resigned in 1891 because of ill-health.

On June 4, 1862, Dr. Boardman married Miss Fannie S. Miller, of New Orleans, who died in 1867. He had no children and the only near relatives who survive him are his brother, George D.

Boardman, of Chicago, a niece, Mrs. E. S. Warren, of Buffalo, and a nephew, Henry F. Boardman, of Troy. The funeral was held July 12th, at which Rev. John M. Brayton, of Corfu, officiated.

The honorary pall-bearers were: Drs. C. C. Wyckoff, Thomas Lothrop, H. R. Hopkins, C. G. Stockton, G. L. Brown and G. W. York, and the active pall-bearers were Drs. D. H. Sherman, J. W. Putnam, DeLancey Rochester, Irving M. Snow, S. Y. Howell, F. Park Lewis and Messrs. Charles Clifton and H. C. Harrower. The interment was at Forest Lawn.

## Figures and Tables

**Figure f1:**